# High Seroprevalence of Feline Leishmaniosis (FeL) in Campania (Italy) Region: Current Epidemiological Scenario

**DOI:** 10.3390/pathogens14121194

**Published:** 2025-11-23

**Authors:** Valentina Foglia Manzillo, Ines Balestrino, Gaetano Oliva, Roberta Brunetti, Stefania Cavallo, Rosa D’Ambrosio, Roberta Pellicanò, Luisa Spadari, Lorella Barca, Federica Bruno, Maria Ortensia Montella, Maria Paola Maurelli, Nunzia Florindo, Manuela Gizzarelli, Mariele De Santi, Loredana Baldi

**Affiliations:** 1 Department of Veterinary Medicine and Animal Productions, University of Naples Federico II, Via Federico Delpino 1, 80137 Naples, Italygaeoliva@unina.it (G.O.); mariaortensia.montella01@universitadipavia.it (M.O.M.); mariele.desanti@unina.it (M.D.S.); 2 Experimental Zooprophylactic Institute of Southern Italy (IZSM), Via Salute 2, 80055 Portici, Italy; 3 National Reference Center for Leishmaniosis (C.Re.Na.L.), OIE Leishmania Reference Laboratory, Experimental Zooprophylactic Institutes of Sicily (IZS Sicilia), Via Gino Marinuzzi 3, 90129 Palermo, Italy; 4 Local Health Authority (ASL) Napoli 3 Sud, U.O.S. Clinica Veterinaria, Via Calastro 26, 80059 Torre del Greco, Italy

**Keywords:** leishmania, epidemiology, cat, IFAT

## Abstract

Feline leishmaniosis (FeL) is still considered an emerging and neglected disease. Cats, once considered accidental hosts, are now recognized as adjunctive reservoirs of the disease, especially in areas where canine (CanL) and human (HumL) leishmaniosis are widespread. Although often asymptomatic, infected cats could contribute to the transmission cycle of the parasite. Recent studies in Campania (Italy) have found a significant prevalence of feline infection, indicating the need to implement diagnostic and surveillance protocols to prevent the spread of the disease. The aim of the study was to outline the current scenario by studying the prevalence of FeL in Campania to identifying the potential zoonotic risk and in addition to validate the Immunofluorescence Antibody Test (IFAT) method for the diagnosis of leishmaniosis in cats. The study involved initially 702 cats; for each cat, a clinical record was compiled, including identification data, anamnesis, and clinical findings. Due to incomplete information, statistical analysis was performed only on a subset of 601 cats. A blood sample was collected to obtain serum/plasma specimens. When feasible, a lymph node fine-needle aspiration was performed. The observed seroprevalence rate was 32.1% (193/601), with a higher seroprevalence in outdoor cats and the presence of asymptomatic seropositive animals (28.0%;54/193), suggesting that felines may act as silent reservoirs of *Leishmania infantum.* An excellent result was obtained for the validation and standardization of the analytical IFAT method for the diagnosis of feline leishmaniasis; therefore, an inter-laboratory test has been carried out to establish the dilution cut-off at ≥1:80 as compatible with infection. Furthermore, a xenodiagnosis examination was conducted on a cat that was infected to more accurately evaluate the possibility of asymptomatic cats acting as carriers of the infection; however, this test resulted negative.

## 1. Introduction

Leishmaniosis is a zoonotic parasitic disease caused by protozoa of the genus *Leishmania*, transmitted by blood-feeding insects of the genus *Phlebotomus* in the Old World. Globally, it ranks as the third most significant vector-borne disease, following malaria and lymphatic filariasis [1]. (While dogs are widely recognized as the principal domestic reservoir of *Leishmania infantum,* infection has been identified in a range of other animal species, such as cats, foxes, jackals, wolves, rodents, horses, mules, cattle, and goats [2,3,4]. In recent years, feline leishmaniosis (FeL) has emerged as a noteworthy and under-recognized disease. Cats are susceptible to the same *Leishmania* species that infects dogs and humans, yet infection in cats is often asymptomatic or subclinical, further complicated by comorbidities and the absence of species-specific diagnostic assays [5]. Despite a growing number of reported cases and an uptick in international research, available data on feline infections remain limited in comparison to canine cases. The epidemiological significance of cats in the transmission of *Leishmania* spp. remains a matter of debate. Some studies assert that cats are rare and relatively resistant hosts, with limited impact on public health [1,6]. Conversely, a cross-sectional study in Córdoba (Spain) documented an overall positivity rate of approximately 30% in both stray dogs and cats via serology and/or Real-Time (qPCR). This research demonstrated that cats develop parasite-specific humoral and cellular responses, though typically to a lesser extent than dogs, suggesting that cats are exposed hosts and may contribute to local endemicity [7] Recent reviews compiling data on alternative vertebrate hosts highlight that domestic cats are consistently detected as infected across Europe and the Mediterranean, suggesting that they may act as secondary or cryptic reservoirs under certain ecological conditions [4,8]. Additionally, high rates of anti-saliva antibodies and seroprevalence in stray cats suggest a potential role as sentinels for vector activity and local transmission intensity, which may inform targeted interventions for both humans and animals [9]. Nonetheless, further studies are required to clarify the precise role of cats in the transmission cycle [3,10]. The Indirect Fluorescent Antibody Test (IFAT) is currently considered a reference test for serological diagnosis, with reported sensitivity around 92% [11]. Latent class Bayesian analysis has shown that IFAT correctly classified approximately 92% of true cases in endemic areas, supporting the adoption of standardized IFAT protocols for feline studies [12]. Additionally, a universally accepted diagnostic protocol is lacking; although IFAT serves as the reference method for serological diagnosis, it has not been fully validated in felines, and the diagnostic cut-off remains to be definitively established [13].

To accurately assess the role of cats in the epidemiological cycle of leishmaniosis, further research utilizing xenodiagnosis and vector infection testing is necessary to better determine their natural reservoir involvement and modes of interspecies transmission [14,15]. The objective of the present study was to evaluate the presence of anti-*Leishmania infantum* antibodies in cats from endemic areas of southern Italy, with particular attention to the identification of seropositive subjects using standardized IFAT protocols.

## 2. Materials and Methods

### 2.1. Study Design and Population

The study was conducted in the Campania region, a historically endemic area for canine and feline leishmaniosis. The most populated provinces of Campania (Napoli and Salerno) were selected for this 3-year program, with few feline samples received from the other 3 provinces (Caserta, Benevento and Avellino). The data collected were organized and visualized through graphs, tables and cartographic representation using the Geographic Information System software (GIS; version QGIS Desktop 3.34.11), to assess the seroprevalence of FeL in that area. The minimum sample size was calculated based on literature data [13]. to ensure the statistical representativeness of the subjects included in the study, considering the following parameters: an expected prevalence of 25% (referring to the spread of *Leishmania infantum* in Southern Italy), a diagnostic sensitivity of 96% and specificity of 98% for the IFAT [16]) and a 95% confidence interval with a significance level of α = 0.05. The feline population to be subjected to diagnostic tests was recruited by passive surveillance (clinically suspicious subjects) and active surveillance (subjects enrolled during the sterilization campaigns of feline colonies) with the involvement of veterinarians, both freelancers and Local Health Authority (ASL) veterinarians. Cats with severe clinical pictures requiring drug therapy, and those with a recent history of immunosuppressive drug treatment, were not enrolled to avoid possible false negative results. For each enrolled cat, a structured clinical record was completed, divided into several sections. This included the veterinarian’s data (private practitioner or ASL employee), the owner/keeper’s data (including residential or holding addresses), and the animal’s identifying information (microchip, name, sex, breed, year of birth, size, coat).

Additional historical data were collected on housing conditions at night, cohabitation with other animals, origin, antiparasitic treatments, and travel history. The record also included the registration of observed clinical signs, potentially consistent with feline leishmaniosis: lymphadenopathy, furfuraceous dermatitis, alopecia, gingivitis/stomatitis, anorexia, depression/weakness, gastrointestinal signs, ulcers, onychopathies, epistaxis, mucosal pallor, weight loss, and ocular lesions. Blood samples were collected from all the cats enrolled in the study to obtain serum and/or plasma specimens. Seventy-nine fine-needle aspirations of lymph nodes were also performed. The samples collected were analyzed by IFAT and Real-Time PCR (qPCR), and the results were subjected to statistical analysis to identify any correlations between risk factors and serological and molecular outcomes; an initial map of the distribution of FeL in Campania was created as a preliminary step towards developing a risk map.

In addition, a Ring Test was performed between the three participating laboratories (Department of Veterinary Medicine (DVM), Naples, Italy; Istututo Superiore di Sanità (ISS), Rome, Italy; Istituto Zooprofilattico del Mezzogiorno (IZSM), Portici, Italy) and the National Reference Centre for Leishmaniosis, Istituto Zooprofilattico della Sicilia, Palermo, Italy (C.Re.Na.L.) for the validation of the IFAT method. Furthermore, a xenodiagnosis examination was conducted on a cat that was infected to more accurately evaluate the possibility of asymptomatic cats acting as carriers of the infection. The study conducted in the three-year period 2022/2024 involved a total of 702 cats, exceeding the calculated minimum sample size of 203.

#### Ethical Statement

All owners were previously informed and gave their consent for treatment, sampling, and data recording. All applicable international, national, and/or institutional guidelines for the care and use of animals were followed (protocol number of ethical clearance: 8126–2022).

### 2.2. Sample Processing

Blood samples (≈2 mL) were collected from the cephalic or jugular vein using sterile disposable syringes with 22 G needles. Samples intended for serum were transferred into tubes with separating gel, while those intended for plasma into K_2_-EDTA tubes, respecting the appropriate ratio between anticoagulant and blood. Tubes were centrifuged under standard conditions, and the obtained serum/plasma was stored until further analysis. Each sample was labeled with a unique identification code corresponding to each animal. Centrifugation of the samples was then performed at the outpatient facility, at a speed of 1300–2000 rpm (RCF) for 10 min at 20–25 °C. The serum/plasma obtained was transferred to 1.5 mL Eppendorf^®^ tubes, marked with the unique code assigned to the sampled subject. Fine needle aspiration (FNA) of peripheral lymph nodes was performed using 22–25 G needles, with or without aspiration depending on lymph node size and vascularization. The aspirated material was retained in the syringe and immediately stored under sterile conditions for subsequent DNA extraction and qPCR analysis. Consequently, the samples were stored at −20 °C until sending to the Laboratory of IZSM for PCR analysis.

### 2.3. Real-Time PCR

The diagnosis of the disease was performed by detecting parasite DNA in lymph node aspirates using qPCR. This test was carried out on a LightCycler^®^ 96 (Roche Life Science, Basel, Switzerland). For the simultaneous detection and quantification of *L. infantum* kinetoplast minicircle DNA, qPCR was performed using the primers LEISH-1 (5′-GGCGTTCTGCGAAAACCG-3′), LEISH-2 (5′-AAAATGGCATTTTCGGGCC-3′), and the TaqMan probe 5′FAM-TGGTGCAGAAATCCCGTTCA-3′-BHQ1. qPCR amplification was conducted in 20 μL reactions containing (final concentrations) SsoAdvanced Universal Probes Supermix (Biorad, Hercules, CA, USA), 0.3 μM of each primer, 0.25 μM of the QLeish probe, and 2 μL of extracted DNA at 10 ng/μL. The cycling conditions were set as follows: UNG step at 50 °C for 150 s, initial denaturation at 95 °C for 10 min, followed by 40 cycles of denaturation at 95 °C for 15 s and annealing/extension at 60 °C for 35 s. A standard curve was constructed using *L. infantum* parasite DNA serially diluted tenfold, ranging from 1 × 10^6^ to 1 parasite per mL. For each sample, a cycle threshold (Ct) value was calculated by determining the point at which fluorescence exceeded the threshold limit. A standard curve was obtained by plotting the Ct values against each parasite DNA standard of known concentration. Samples showing a parasite load of ≥1 parasite per mL were considered positive.

### 2.4. Immuno-Fluorescence Antibody Test (IFAT)

The serological examination included IFAT assays, according to the protocol of the Office International des Epizooties (WOAH) Terrestrial Manual (sensitivity 0.96, specificity 0.98). The procedure was performed using multispot slides containing wells coated with *L. infantum* promastigote antigens. A positive control (serum from cats with confirmed specific antibody activity) and a negative control (a pooled serum from cats certified negative for anti-*Leishmania* antibodies) were included in each run. Sera were prepared by serial doubling dilutions (from 1:40 to 1:5120 for feline samples) in phosphate-buffered saline (PBS, pH 7.2) and added to the antigen-coated wells. IFAT slides were then incubated for 30 min at 37 °C before proceeding with the subsequent steps. The slides were subjected to three consecutive washes in PBS (10 min each), incubated with fluorescein-labeled goat immunoglobulins (anti-cat, anti-dog, and anti-human IgG, Sigma Aldrich, Saint Louis, MO, USA) and incubated at 37 °C for 30 min. The slides were washed three times (10 min each) in PBS, and the reactivity of the sera was detected using a Leica DM 4000B fluorescence microscope (Leica, Heerbrugg, Switzerland) at 40× magnification.

#### IFAT Cut-Off Values Update

In addition to the standard IFAT procedure, updated cut-off values were adopted to improve the diagnostic accuracy in cats, based on recent validation data. The C.Re.Na.L., WOAH Reference Laboratory, has recently proposed updated cut-off values for feline IFAT in response to the revision of Chapter 3.1.11 of the WOAH Terrestrial Manual (expected adoption May 2026). Based on current evidence, serum titers of 1:40 are considered indicative of suspected exposure, while titers ≥ 1:80 are regarded as positive and compatible with infection.

### 2.5. Ring Test

To standardize the diagnostic IFAT method and ensure the reproducibility and reliability of IFAT results for FeL, an interlaboratory ring test was performed. The laboratories, identified as PT 16/24 Leish, PT 17/24 Leish, and PT 18/24 Leish, tested a panel of 10 feline serum samples previously analyzed at the C.Re.Na.L. The samples were divided into two categories according to their serostatus. In addition, each laboratory received aliquots of positive and negative Reference Material (RM), with the positive RM titrated at 1:2560.

To assess the degree of concordance under reproducibility conditions, the K index was calculated between the participating laboratories and the C.Re.Na.L.; the acceptability criterion was established when the value of K is ≥0.61 (good level of agreement).

### 2.6. Xenodiagnosis

To identify the ideal candidate for xenodiagnosis, several clinical cases presented at the Federico II Department of Veterinary Medicine, Teaching Hospital (TH) were analyzed. Finally, a European, neutered male cat of about two years of age, was selected. The cat was visited by a veterinary practitioner for chronic lameness and lymph node enlargement. During the clinical examination, no significant abnormalities were found, except for lymphadenopathy of both popliteal lymph nodes. Abdominal ultrasound and X-rays ruled out involvement of other organs or structures. Laboratory tests showed an increase in total serum protein (9.20 g/dL; reference range: 5.8–7.8 g/dL) with hypergammaglobulinemia (5.9 g/dL; reference range: 0.7–1.6 g/dL), IFAT demonstrated a titer of 1:160 while it was negative to Feline Immunodeficiency Virus (FIV) and Feline Leukemia Virus (FeLV) tests (Idexx Snap^®^ Combo plus). The cat was treated with meloxicam 0.1 mg/kg/die (then tapered to 0.05 mg/kg/die) for seven days. After two weeks, the cat was referred to TH.

During the clinical examination, the cat showed no symptoms as the lameness and lymph node enlargement had disappeared. During the visit, bone marrow and lymph node samples were taken for qPCRmolecular testing [17]. in the Parasitology and Parasitic Diseases Laboratory of the Department of Veterinary Medicine to confirm the presence of the pathogen. In addition, a sample of bone marrow and lymph node material was sent to the ISS Unit for *Leishmania* spp.- culture analysis. The results of both tests were negative, while the IFAT serological test continued to be positive for the presence of anti-*Leishmania* spp. antibodies.

Xenodiagnosis was performed in collaboration with the ISS, which provided sand flies. Briefly, a cohort of 100 female sand flies from the *Phlebotomus perniciosus* colony of ISS was used, all confirmed negative for infection prior to the experiment. Additionally, 10% male flies were included to potentially enhance feeding activity. After owner consent, the cat was sedated using the association of alphaxalone (2 mg/kg) and butorphanol (0.2 mg/kg), and exposed to the bite 4–6-day-old, sugar-fed sandfly females, in a fine net cage. After 90 min exposure, both dead and alive sand flies were collected by a mouth aspirator and pooled in cylindrical plastic pots (400 mL) fitted with a tight lid, provided with a piece of cotton soaked with glucose-saturated solution, and maintained thereafter at the usual rearing conditions. Sand flies were examined individually after 24 h post-exposure to identify the blood engorged, and 96 h after to assess the presence of *Leishmania* spp. promastigote [18].

### 2.7. Statistical Analysis

Statistical analyses were performed using R software (version 4.5.1). Demographic, clinical, and management characteristics of the study population were described by stratifying subjects according to *Leishmania* spp. test positivity.

For each categorical variable, absolute and relative frequencies were reported. Differences between groups (positive vs. negative) were assessed using Fisher’s exact test. Subsequently, to estimate the association between predictive factors and positivity to feline leishmaniosis, a logistic regression model was applied. Before model creation, each variable was transformed into a factor (No = 0, Yes = 1). Estimates were obtained by maximum likelihood. For each variable, the following were calculated: (i) Odds ratios (OR) (exponentiated estimated coefficients), (ii) 95% confidence intervals (95% CI), (iii) *p*-values based on Wald’s test.

To assess multicollinearity among predictors included in the logistic regression model, the Variance Inflation Factor (VIF) was calculated. A VIF > 5 was considered indicative of potential collinearity, while values > 10 were interpreted as strong collinearity. In addition, a deviance analysis (ANOVA) with a Chi-square test was performed to estimate the statistical contribution of individual variables to the overall model.

### 2.8. Spatial Representation of Data

Information with a spatial location can be processed using GIS. In this study, data relating to the origin of the samples and the spatial distribution of the results were managed from a geographical perspective. All processing was carried out using QGIS Desktop 3.34.11 software.

## 3. Results

### 3.1. Overall Distribution of Cases

In order to describe the trend of outcomes in the period under review, the data collected were analyzed considering both the temporal distribution (by year) and their distribution according to sex and geographical location. It is important to underline that clinical records were not always provided or completed correctly; therefore, statistical analysis was performed only on a subset of 601 cats.

#### 3.1.1. Distribution by Years

In the three-year period considered (2022–2024), a total of 601 cases were analyzed. Of these, 408 (67.9%) had a negative result, while 193 (32.1%) had a positive result. The annual distribution showed a substantial increase in the number of cases between 2022 (105 total) and 2023 (317 cases), followed by a reduction in 2024 (179 cases). In each year, negative outcomes accounted for the prevailing share, ranging from 58.1% in 2022 to 69.7% in 2023, up to 70.4% in 2024 (Table 1). The Chi-square test of independence did not reveal statistically significant differences in prevalence among the three years (χ^2^ = 5.62; df = 2; *p* = 0.060).

#### 3.1.2. Distribution by Gender

**Figure 1:**
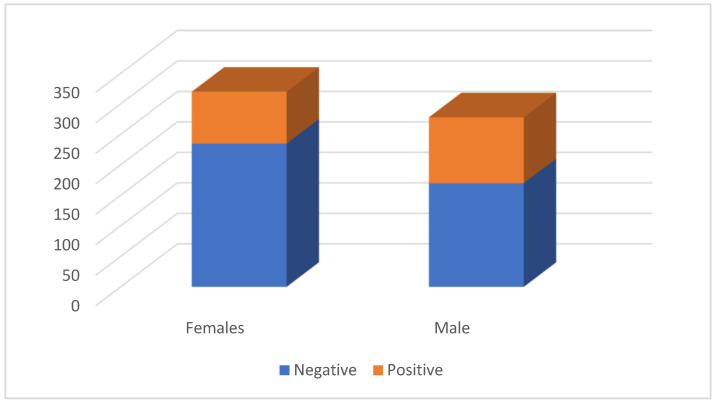
Distribution of the number of cats by outcome and sex.

Of the cases for which sex information was available (598/601; 99.5%), 320 (53.5%) involved females and 278 (46.5%) males. Positive and negative results according to gender are shown in Figure 1.

#### 3.1.3. Distribution Among the Five Campania Region Provinces (Napoli, Salerno, Benevento, Caserta, Avellino)

The distribution of enrolled cases was not homogeneous. Most of the cases were obtained from Napoli (463/601; 77.0%), followed by Salerno (99; 16.5%), Benevento (15; 2.5%), Avellino (9; 1.5%) and Caserta (5; 0.83%) ASL. Ten cases were collected outside the region (10; 1.7%).

**Figure 2:**
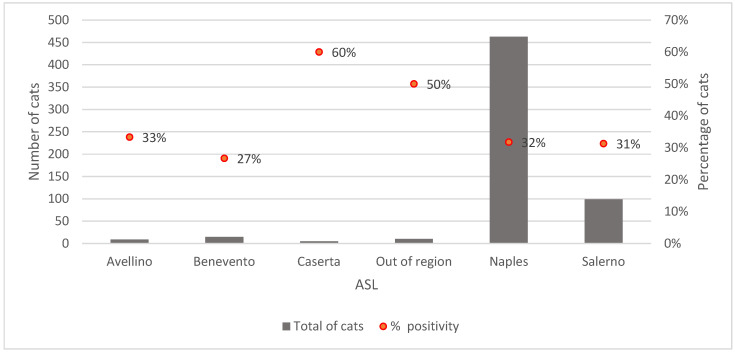
Distribution by ASL: number of cats and percentage of FeL positivity.

The proportion of positive outcomes showed territorial variability: Caserta recorded the highest share (60.0%; 3 out of 5 positives), followed by cases outside the region (50.0%; 5 out of 10 positive), Avellino (33.3%; 3 out of 9 positive), Napoli (31.8%; 147 out of 463 positive), Salerno (31.3%; 31 out of 99 positive), and finally Benevento (26.7%; 4 out of 15 positive), which showed the lowest percentage (Figure 2).

### 3.2. Statistical Analysis Results

Statistical analysis was performed to assess potential associations between seropositivity and variables such as age, sex, lifestyle (indoor/outdoor), and clinical signs. Statistical analysis among the five Campania Provinces was not performed due to the limited number of samples available for Avellino, Caserta and Benevento Provinces.

The minimum sample size was estimated a priori at 273 feline subjects. This calculation was made to ensure adequate statistical power (α = 0.05, power = 0.80) to detect clinically relevant differences in the main predictors. Due to the lack of demographic information, no stratification of the population was possible. Nevertheless, the analyzed sample exceeded the minimum estimated size, with 601 cats included in the study.

Table 2 summarizes all the data obtained from the clinical and anamnestic records, along with the IFAT-based classification into positive (P) and negative (N) categories, and the relative *p*-value.

From Table 2, it emerges that males were significantly more represented in the positive group compared to females. Weight loss was more frequent in positive subjects (17.6%) compared to negatives (9.6%), as were other clinical signs such as mucosal pallor, ocular lesions, and gingivitis. Most subjects were stray, originated from Napoli, lived outdoors, and cohabited with other animals.

Most clinical variables did not show significant differences between groups, except for sex and weight loss, which were significantly associated with the outcome.

The logistic regression model was built using only the variables that were statistically significant in Fisher’s test, with the addition of the covariate “ocular lesions,” whose *p*-value (0.06) was close to the 0.05 significance threshold.

According to the multivariate logistic regression analysis, the odds ratios and confidence intervals indicate that:

○ Males had an odds ratio of 1.67 (95% CI: 1.18–2.38; *p* = 0.0039), indicating a significantly higher likelihood of contracting the disease compared to females.
○ Weight loss was associated with an odds ratio of 1.91 (95% CI: 1.15–3.17; *p* = 0.012), suggesting that subjects with weight loss had almost double the odds of being positive compared to those without.
○ Ocular lesions showed an odds ratio of 1.72 (95% CI: 0.79–3.71; *p* = 0.164), indicating a non-significant association, as the confidence interval included 1. This finding is consistent with Fisher’s test.

VIF values for all variables were below 2 (Sex = 1.01; Weight loss = 1.01; Ocular lesions = 1.01), suggesting no multicollinearity issues in the model.

The deviance analysis confirmed that including sex and weight loss significantly reduced residual deviance (*p* = 0.00135 and *p* = 0.00949, respectively), whereas ocular lesions did not significantly improve the model (*p* = 0.167).

These results suggest that male cats and those experiencing weight loss have a higher likelihood of being IFAT-positive, while other clinical and demographic factors do not appear to be strong predictors.

### 3.3. GIS Analysis

**Figure 3:**
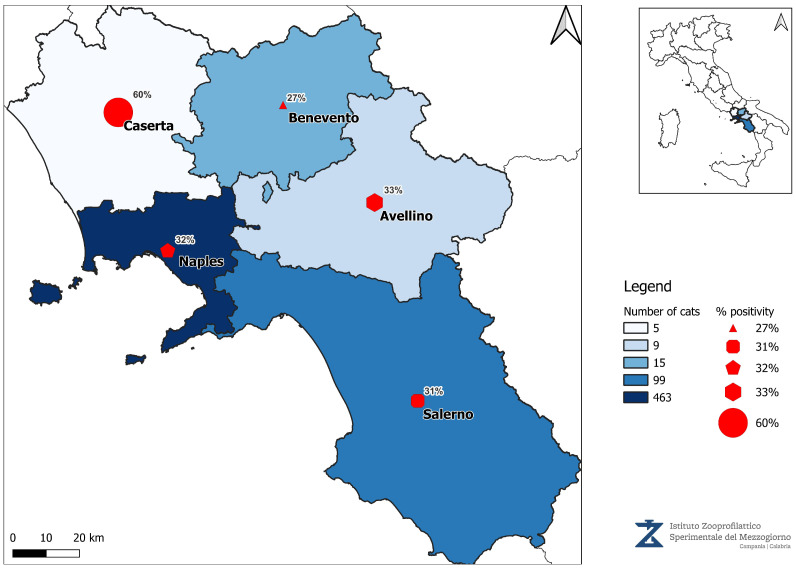
Map of the number of samples received and IFAT positive results.

The current distribution of *Leishmaniosis* in Campania can be visualized in the map below, highlighting the ongoing public health challenges in Southern Italy (Figure 3).

### 3.4. qPCR Results

qPCR diagnostic investigations were performed on 79 samples, 6 of which were positive (7.6%), all participating to a sterilization program planned by the municipality of Nocera Inferiore (Salerno). In total, 16 of these 79 cats tested positive for IFAT, 3 of which were positive to qPCR too. Unfortunately, since these were stray cats, it was not possible to recapture any specimens in order to carry out in-depth analyses.

### 3.5. Ring Test Results

The three participating laboratories, identified as PT 16/24 Leish, PT 17/24 Leish and PT 18/24 Leish, achieved concordant results, meeting the minimum criterion of acceptability (K value) for inter-laboratory agreement (Table 3).

The level of agreement was assessed using K value ≥ 0.61, considered indicative of “good” to “very good” concordance according to Landis and Koch’s classification.

This outcome demonstrates that the IFAT assay produced reproducible results across different laboratories, ensuring the reliability of the diagnostic method. The successful validation of the interlaboratory ring test therefore supports the robustness of IFAT standardization and strengthens its potential application as a reference serological tool for FeL diagnosis.

### 3.6. Xenodiagnosis Results

The overall feeding rate observed was 32% (32 out of 100 employed female sandflies). Upon microscopic examination of the engorged females, no evidence of infection was detected.

## 4. Discussion

Leishmaniosis in cats is emerging as a noteworthy concern, particularly in regions endemic for *Leishmania infantum*. Historically, domestic cats (*Felis catus*) have been regarded as atypical hosts for *Leishmania* spp., with a presumed high resistance possibly linked to genetic factors [19]. However, recent advances in feline medicine and the development of sensitive serological and molecular assays have revealed that infection in cats may be underestimated. While cats have long been considered accidental hosts, more recent epidemiological data increasingly implicate them as potential alternative reservoirs for the parasite [4,8]. This shift in perspective is especially relevant in highly endemic areas of southern Italy such already burdened by CanL and HumL. The geographical overlap of FeL, CanL, and HumL suggests that cats may contribute to the transmission cycle of the parasite.

This study provides recent information on the exposure of cats to *Leishmania infantum* in southern Italy, confirming that cats living in endemic areas can show measurable antibody responses. The total seroprevalence, equal to 32.1%, is in line with previous studies from Mediterranean areas, which generally range between 10% and 35% depending on diagnostic methods and populations analyzed [1,20,21]. These data reinforce the idea that cats can serve as indicators of the presence of *L. infantum* in the environment. The findings reveal a notably high seroprevalence in cats, suggesting that felines may play a more substantial role in the epidemiology of leishmaniosis than previously recognized. This shifts the current understanding of the disease’s epidemiology and offers valuable insights for developing more effective control strategies in both veterinary and public health contexts. However, IFAT cross-reactivity among different *Leishmania* species represents a significant diagnostic challenge. A recent study [15] reported a critical finding: cats were screened for leishmania infection using IFAT with promastigotes of both *L. infantum* and *L. tarentolae*—a non-pathogenic reptile-specific species recently identified in cats—and importantly, 18.2% of infected cats tested positive for both species simultaneously by IFAT, despite species-specific differences detected by molecular methods.

Among risk factors, male sex was significantly associated with seropositivity, in agreement with previous research. Behaviors and ecological factors, such as outdoor exposure, may increase contact with vectors, thereby increasing the risk of infection. Weight loss also showed a significant link with positive IFAT results, which could indicate potential chronic infection or co-infections that compromise immune function. A study performed on a low number of cats from the same Region [22] may indicate that immunosuppressive diseases such as FeLV and FIV may be associated with FeL. In our study, due to a high number of samples, it was not possible to test these two viral infections for economic reasons. Other clinical symptoms, such as eye lesions and pale mucous membranes, were found more frequently in seropositive cats, although without statistical significance, possibly due to incomplete clinical data.

The proportion of qPCR-positive samples (7.59%) in serologically positive cats was similar to previous studies [23,24]. In our study, the evidence of *Leishmania* DNA in lymph node aspirates confirms the vascularization systemic dissemination of the parasite. The limitation of this technique in cats is due to the necessity to sedate the animal, suggesting the requirement for further studies on less invasive procedures for DNA detection. In addition, the impossibility of recapturing many stray cats limited further follow-up.

The interlaboratory test performed in the present study showed a good level of agreement (K ≥ 0.61), validating the robustness of the standardized IFAT protocol. This result was significant considering the persistent problem of diagnostic variability in feline leishmaniosis. Homogeneous IFAT procedures, including standardized antigen preparation and cut-off thresholds, could improve comparability between studies and strengthen the reliability of surveillance.

No evidence of parasite transmission was found in xenodiagnosis, as none of the fed sand flies became infected. Although this result may be influenced by a modest feeding rate obtained in a mildly symptomatic positive cat, not coinfected with immune-unbalancing viruses, it may indicate that a clinically healthy infected cat could have low competence as a reservoir for *L. infantum*. There is much evidence that demonstrates the role of cats as a reservoir for *L. infantum* as assessed by xenodiagnoses studies both in Europe [25] and in Brazil [26,27,28] performed with *Phlebotomus pernicious* and *Lutzomya longipalpis,* respectively. The experimental transmission of *L. infantum* from a naturally infected cat to a healthy domestic dog has also been demonstrated [29]. However, more accurate xenodiagnosis studies are necessary in a larger number of animals, with different clinical presentations, also considering that preventive measures remain inadequate, partially due to the toxicity of common repellents for dogs when used in cats [30].

This study has some limitations, including the lack of complete clinical data and a rather small number of samples analyzed by qPCR. Despite this, the overall sample size proved to be adequate to ensure the statistical robustness of the analyses performed. Furthermore, the integration of serological, molecular, and entomological data provided a more detailed picture of the exposure of cats in the area under investigation.

## 5. Conclusions

In summary, cats from endemic regions of southern Italy show significant levels of exposure to *L. infantum*, particularly among males and those that are underweight. The validated IFAT protocol confirmed its repeatability in the various laboratories involved, proving to be suitable for large-scale investigations. Due to the confirmed role of the cat as a *Leishmania infantum* reservoir, monitoring this domestic animal can be an effective early warning system for the local spread of the parasite, thus contributing to integrated leishmaniosis control strategies.

## Figures and Tables

**Table 1:** Distribution of outcomes by year.

	DISTRIBUTION OF OUTCOMES BY YEAR				
	2022	2023	2024	Total	%
**NEGATIVE**	61	221	126	408	67.89%
**POSITIVE**	44	96	53	193	32.11%
**TOTAL**	105	317	179	601	100%

**Table 2:** Data obtained from the clinical and anamnestic records, along with the IFAT-based classification into positive (P) and negative (N) categories, and the relative *p*-value.

Variable	N (N = 408)	P (N = 193)	*p*-Value
Status			0.198
Pet-owned	21 (5.1%)	5 (2.6%)	
Stray	387 (94.9%)	188 (97.4%)	
Sex			<0.05
Female	235 (57.6%)	85 (44.0%)	
Male	170 (41.7%)	108 (56.0%)	
Missing	3 (0.7%)	0 (0%)	
Nighttime Shelter			0.669
Indoors	5 (1.2%)	1 (0.5%)	
Outdoors	381 (93.4%)	188 (97.4%)	
Missing	22 (5.4%)	4 (2.1%)	
Living with other animals			0.49
No	32 (7.8%)	11 (5.7%)	
Yes	281 (68.9%)	131 (67.9%)	
Missing	95 (23.3%)	51 (26.4%)	
Antiparasitic treatments			0.398
No	242 (59.3%)	102 (52.8%)	
Yes	9 (2.2%)	6 (3.1%)	
Missing	157 (38.5%)	85 (44.0%)	
Clinical symptoms			0.135
No	140 (34.3%)	54 (28.0%)	
Yes	268 (65.7%)	139 (72.0%)	
Lymphadenopathy			0.897
No	355 (87.0%)	169 (87.6%)	
Yes	53 (13.0%)	24 (12.4%)	
Furfuraceous dermatitis			0.839
No	389 (95.3%)	183 (94.8%)	
Yes	19 (4.7%)	10 (5.2%)	
Anorexia			0.34
No	325 (79.7%)	147 (76.2%)	
Yes	83 (20.3%)	46 (23.8%)	
Weakness			0.471
No	259 (63.5%)	116 (60.1%)	
Yes	149 (36.5%)	77 (39.9%)	
Gastrointestinal signs			0.325
No	380 (93.1%)	175 (90.7%)	
Yes	28 (6.9%)	18 (9.3%)	
Ulcers			0.585
No	385 (94.4%)	180 (93.3%)	
Yes	23 (5.6%)	13 (6.7%)	
Onychopathies			1
No	406 (99.5%)	193 (100%)	
Yes	2 (0.5%)	0 (0%)	
Rhinorrhea			0.77
No	399 (97.8%)	187 (97.5%)	
Yes	9 (2.2%)	5 (2.6%)	
Mucosal pallor			0.007
No	303 (74.3%)	130 (67.4%)	
Yes	105 (25.7%)	63 (32.6%)	
Weight loss			<0.05
No	369(90.4%)	159 (82.4%)	
Yes	39 (9.6%)	34 (17.6%)	
Ocular lesions			0.067
No	393 (96.3%)	179 (92.7%)	
Yes	15 (3.7%)	14 (7.3%)	
Alopecia			0.26
No	379 (92.9%)	174 (90.2%)	
Yes	29 (7.1%)	19 (9.8%)	
Gingivitis/Stomatitis			0.268
No	335 (82.1%)	151 (78.2%)	
Yes	73 (17.9%)	42 (21.8%)	

**Table 3:** Interlaboratory IFAT Comparison Results.

Labs	K Value
PT 16/24 Leish	1
PT 17/24 Leish	0.78
PT 18/24 Leish	0.8

## Data Availability

The raw data supporting the conclusions of this article will be made available by the authors on request.
